# Supplementary material for: Comparison of Methods for Incorporating Related Data When Developing Clinical Prediction Models: A Simulation Study
Source: Biom J. 2026 Aug 27;68(5):e70166. doi: 10.1002/bimj.70166 (PMC13520826; doi:10.1002/bimj.70166)
Supplement: Supplementary file 2 [file BIMJ-68-e70166-s001.zip › third submission code review - R2/reproducibility_supplement/results/Plots/combined_plots/indv_stability_plot_combined_no_shift_SC_ID1_TS0.25_DS4.pdf]

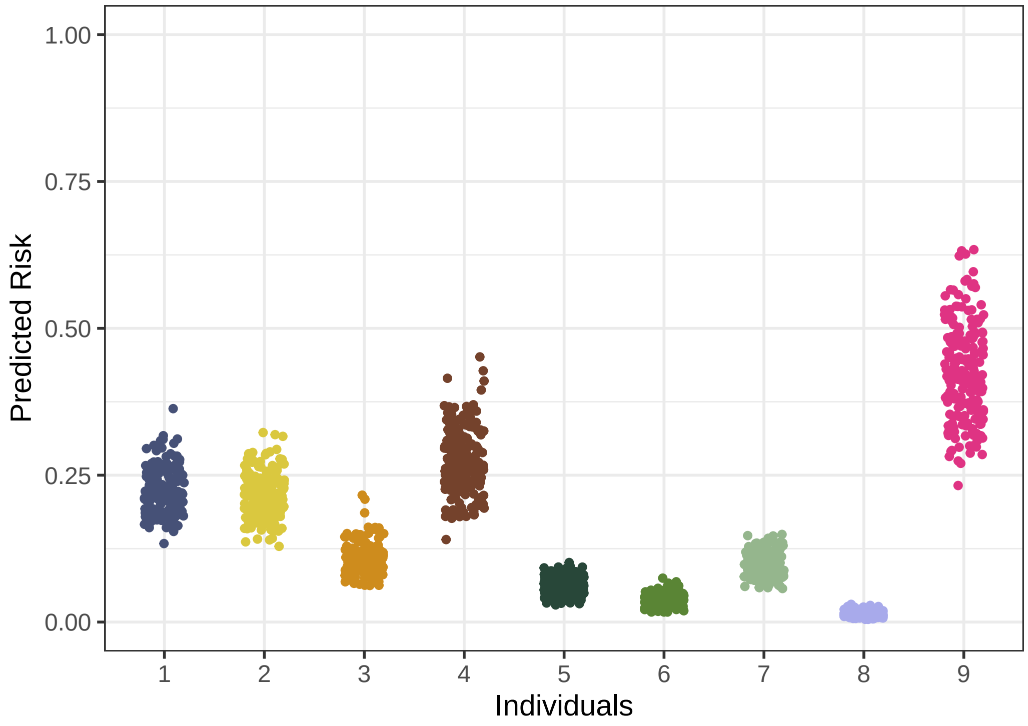

(a) Target−only

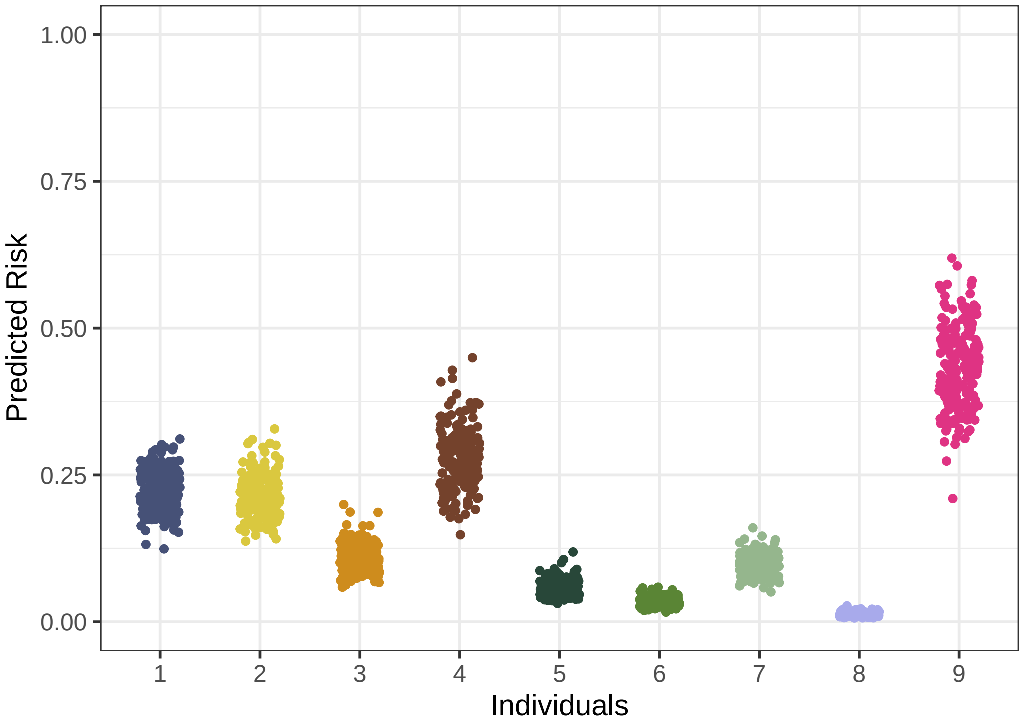

(b) Intercept Recalibration−all data

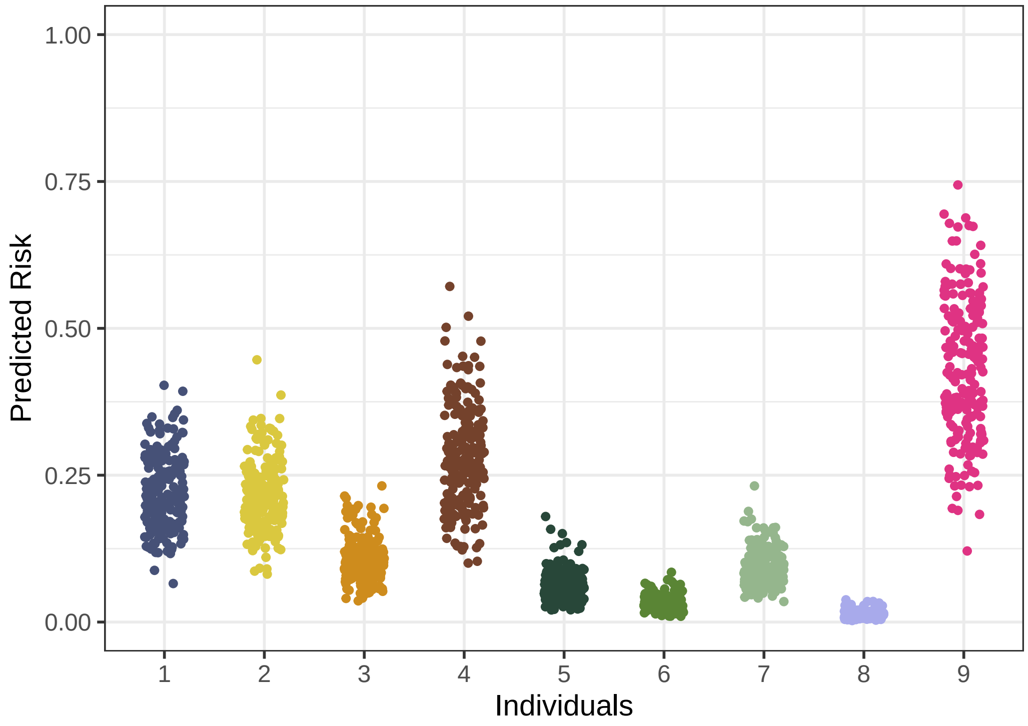

(c) Intercept Recalibration−ancillary only

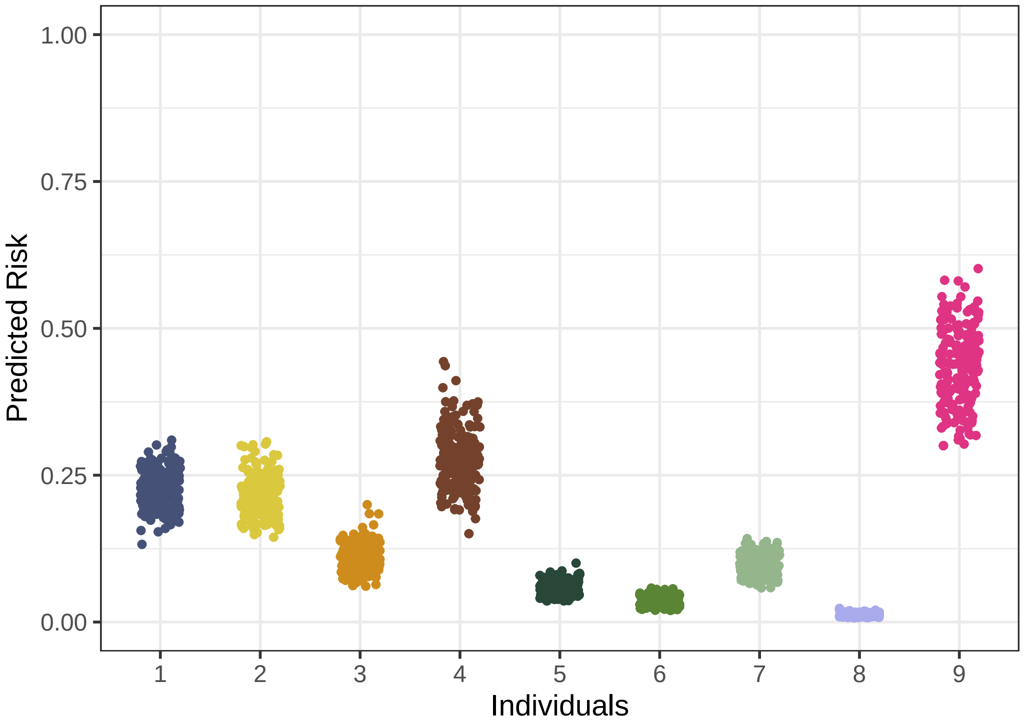

(d) Logistic Recalibration−all data

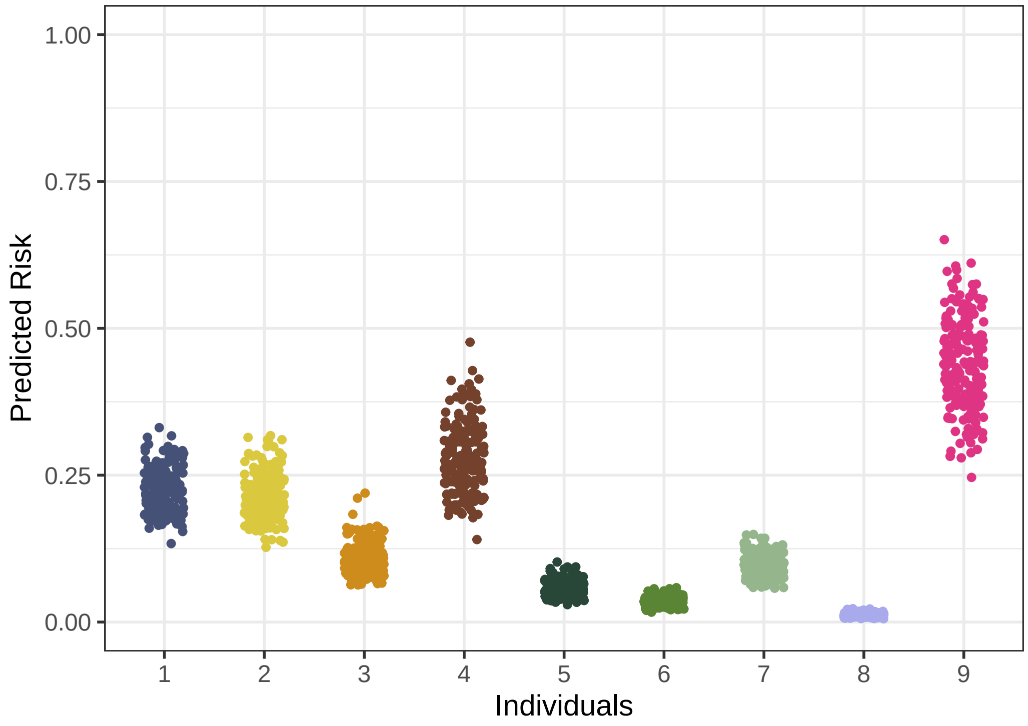

(e) Logistic Recalibration−ancillary only

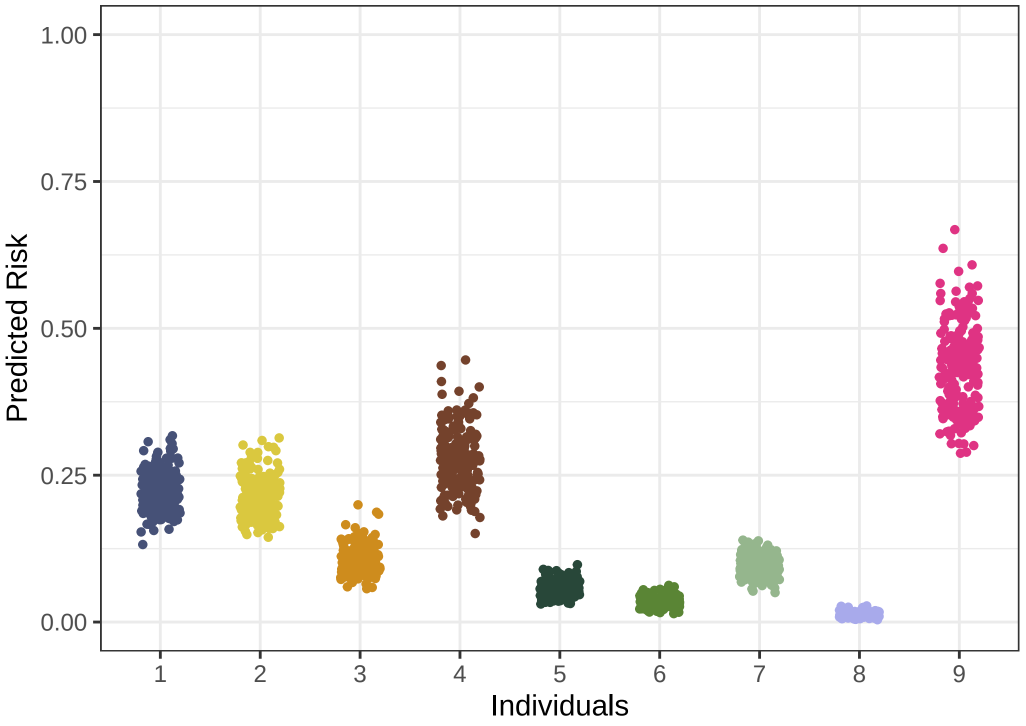

(f) Membership−based recalibration
